# Effect of intravenous hydrocortisone and periarticular triamcinolone acetonide injection on quadriceps strength recovery in patients undergoing total knee arthroplasty: a prospective randomised controlled trial

**DOI:** 10.1186/s43019-026-00328-2

**Published:** 2026-06-30

**Authors:** Kulapat Chulsomlee, Thitiphol Wanitchanont, Chattrapat Asavakunalai, Sorawut Thamyongkit, Nithid Sri-utenchai, Siwadol Wongsak, Chavarat Jarungvittayakon, Paphon Sa-ngasoongsong

**Affiliations:** 1 Chakri Naruebodindra Medical Institute, Ramathibodi Hospital, Samutprakan, Thailand; 2 Department of Orthopedics, Ramathibodi Hospital, Bangkok, Thailand

**Keywords:** Periarticular injection, Intravenous injection, Corticosteroid injection, Total knee arthroplasty, Quadriceps strength, Pain control

## Abstract

**Background:**

Postoperative pain management is crucial for optimising recovery after total knee arthroplasty. Adequate pain control facilitates early rehabilitation, enhances quadriceps muscle recovery, improves knee function and improves patient satisfaction. Corticosteroids are widely used for postoperative pain management. However, studies comparing the efficacy of periarticular (PA) and intravenous (IV) corticosteroid injection are limited. Therefore, this study aimed to evaluate the effect of PA and IV corticosteroid injections on quadriceps strength (QS) recovery in patients undergoing total knee arthroplasty.

**Methods:**

A total of 59 patients were included in this study. Patients were randomly assigned to two groups: the PA triamcinolone group (*n* = 29) and the IV hydrocortisone group (*n* = 30). Patients were followed up for 6 months and their QS, Visual Analogue Scale score, Modified Timed Up and Go (MTUGT) score, Western Ontario and McMaster Universities Osteoarthritis Index score, Knee Society Score and inflammatory marker levels were assessed.

**Results:**

On postoperative day 3, the PA group exhibited a significantly lower reduction in QS (−43.11% ± 23.36% versus −63.7% ± 17.67%, *p* = 0.02) and significantly lower changes in MTUGT scores than the IV group (153.3% versus 301.3%, *p* < 0.01). Additionally, the knee flexion angle was significantly greater in the PA group than in the IV group on postoperative day 3 (91° ± 11° versus 82.6° ± 10.9°, *p* < 0.01) and at week 2 (103.2° ± 12.2° versus 97.4° ± 8.7°, *p* = 0.04). C-reactive protein levels were significantly lower in the PA group than in the IV group on postoperative day 1 (10.4; IQR: 5.3–17.2 versus 15.4; IQR: 9.4–28.6, *p* = 0.01), on day 3 (65.5; IQR: 38.3–96 versus 119.1; IQR: 69.6–146.1, *p* < 0.01) and at week 2 (3.9 versus 8.9, *p* = 0.01). On postoperative day 3, the PA group had significantly higher glucose levels than the IV group (115; IQR: 106–127 versus 106; IQR: 93–122, *p* = 0.02). No wound complications were observed in both groups.

**Conclusions:**

PA corticosteroid injection improves knee function in terms of muscle recovery, faster ambulation, increased knee flexion and reduced inflammation for up to 2 weeks after surgery compared with IV corticosteroid injection. A transient increase in serum glucose was observed but is unlikely to be clinically significant.

## Background

Total knee arthroplasty (TKA) is the standard treatment for end-stage knee osteoarthritis. TKA can help patients regain their ability to perform activities of daily living with improved functional capacity. The functional performance of the knee after surgery is a key indicator of the success of the surgery. Therefore, the rapid recovery of quadriceps strength (QS) is important. Previous studies have shown that QS recovery after TKA may require 6–12 months [1, 2]. Consistent physiotherapy and effective postoperative pain management significantly enhance QS restoration after surgery.

The use of corticosteroids during the perioperative period has been reported to significantly reduce postoperative pain, minimise nausea and vomiting and enable earlier ambulation [3, 4]. Corticosteroids exert anti-inflammatory effects, reducing soft tissue inflammation caused by surgical trauma. Previous studies have shown that patients receiving corticosteroids exhibit significantly lower levels of inflammatory markers, such as C-reactive protein (CRP) and interleukin-6, which contributes to decreased pain and inflammation [5]. Consequently, reduced postoperative pain allows early initiation of gait training and muscle strengthening exercises.

Corticosteroids can be administered intraoperatively via two primary routes: intravenous (IV) and periarticular (PA) injection. Both routes have demonstrated significant benefits in reducing postoperative pain and earlier initiation of physiotherapy and rehabilitation [6–8]. However, no previous studies have directly compared the efficacy of IV corticosteroid administration with that of PA corticosteroid injection.

Therefore, this study aimed to evaluate and compare the effects of these two administration routes in terms of objective clinical outcomes, including QS recovery, time to ambulation and patient satisfaction.

## Materials and methods

This study was approved by the Institutional Ethics Committee (protocol no. COA.MURA2022/692) and was conducted in accordance with the 1964 Declaration of Helsinki and its later amendments. This trial was registered with the Thai Clinical Trials Registry (TCTR20240419006 at thaiclinicaltrials.org). Written informed consent was obtained from all participants before enrolment.

### Trial registration and protocol access

The full trial protocol and Statistical Analysis Plan (SAP) are available from the corresponding author upon reasonable request or may be provided as supplementary material following journal instructions.

### Availability of data and materials

De-identified individual participant data (IPD), data dictionary and statistical analysis code will be available from the corresponding author upon reasonable request, following approval by the institutional ethics committee and completion of a data-sharing agreement.

### Patient and public involvement

Patients and members of the public were not involved in the design, conduct, reporting or dissemination planning of this research.

### Study population

This single-centre, prospective, single-blinded randomised controlled trial included patients scheduled for primary TKA at the institution, between September 2023 and August 2024. The inclusion criteria included (1) patients diagnosed with advanced knee osteoarthritis, (2) age between 55 and 80 years, (3) preoperative QS graded as 5 according to the Medical Research Council Manual Muscle Testing scale [9], (4) independent ambulation with or without gait assistance and (5) ability to comply with the study protocol and provide written informed consent. The exclusion criteria included (1) severe deformity requiring augmentation (metal or bone graft) or a constrained prosthesis, (2) revision surgery or previous surgery on the affected limb, (3) contraindications to nonsteroidal anti-inflammatory drugs or corticosteroids, (4) diagnosis of inflammatory polyarthritis (rheumatoid arthritis or gout), (5) uncontrolled diabetes mellitus and (6) concomitant lower extremity or neuromuscular disorders.

### Subject allocation and randomization

Randomisation was performed using STATA version 18 (StataCorp LLC, College Station, TX, USA) with a block size of four. The allocation was concealed using sequentially numbered, sealed opaque envelopes. Participants were randomly assigned in a 1:1 ratio to either the IV corticosteroid (IV group) or PA corticosteroid (PA group) injection group. Group allocation and drug preparation were performed by a research assistant who was not involved in outcome assessments on the day of hospital admission.

Patients in the IV group received 100 mg IV hydrocortisone 2 h before surgery, followed by 25 mg IV hydrocortisone every 8 h for 24 h after surgery. Additionally, patients received a PA injection of 20 mL of 0.5% bupivacaine (Marcaine) and 30 mg of ketorolac during the surgery.

Patients in the PA group received a single intraoperative PA injection of 40 mg of triamcinolone acetonide combined with 20 mL of 0.5% bupivacaine and 30 mg of ketorolac, administered into the surrounding soft tissue.

### Surgical procedure

All procedures were performed by a single fellowship-trained arthroplasty surgeon with more than 10 years of experience (KC). All patients received combined regional anaesthesia, including spinal anaesthesia and an adductor canal block. A standard medial parapatellar approach was used, following the principles of mechanical alignment with conventional instrumentation and a measured resection technique. A cemented, fixed-bearing, posterior cruciate-substituting prosthesis was implanted in all patients. Patellar resurfacing was performed in all patients. A pneumatic tourniquet was inflated to 150 mmHg above the patient’s systolic blood pressure and deflated after wound closure.

Procedure fidelity was maintained using a standardized operative checklist and uniform periarticular injection protocol verified by a research assistant not involved in outcome assessment.

A multimodal PA injection cocktail comprising 20 mL of 0.5% bupivacaine (Marcaine) and 30 mg of ketorolac, with or without 40 mg of triamcinolone acetonide, was prepared by a scrub nurse under sterile conditions in a separate area of the operating room. The syringe was concealed with sterile opaque wrapping to maintain blinding. Injection was administered into deep tissue structures only, specifically targeting the posterior capsule and the medial and lateral posterior gutters, following the technique described by Tsukada et al.[10] Care was taken to avoid infiltration into superficial tissues, such as the quadriceps tendon, patellar tendon and subcutaneous tissues adjacent to the incision, to minimise the risk of corticosteroid-related complications. Injection was performed before the final implant insertion. The surgeon, assistant and scrub nurse were all blinded to the contents of the injection throughout the procedure. A drain tube was placed before closure of the joint capsule. The length of the quadriceps tenotomy was measured from the most proximal aspect of the incision to the superior border of the patella. The capsule was sutured with 1–0 Vicryl (Ethicon, Cincinnati, OH, USA), followed by subcutaneous closure using 2–0 Vicryl and 4–0 Monocryl (Ethicon). Skin closure was completed using sterile strips. After capsule closure, 20 mL of tranexamic acid was injected into the joint cavity via the drain tube, which was then clamped for 2 h, following the protocol described by Sa-ngasoongsong et al.[11]

### Postoperative protocol

Postoperative care and rehabilitation were performed according to a standardised protocol administered by a research assistant who was not involved in the statistical analysis. All patients received the same analgesic regimen and rehabilitation programme. Perioperative pain was managed with IV ketorolac administered every 8 h for the first 48 h, along with patient-controlled IV morphine. During hospitalisation, patients were administered oral analgesics, including acetaminophen combined with codeine every 8 h and 75 mg pregabalin at bedtime. Mechanical compression devices, early mobilisation with range of motion (ROM) exercises and low-dose aspirin (100 mg once daily) were used for venous thromboembolism prophylaxis. Patients were encouraged to sit at the bedside, initiate passive ROM exercises and begin ambulation training with a walker within 24 h postoperatively. Blood glucose levels were monitored by point-of-care testing before meals and at bedtime from the day of surgery until discharge. Subcutaneous insulin was administered when the point-of-care glucose level exceeded 200 mg/dL.

Patients were discharged once their pain was well-controlled, they were able to ambulate independently with a walker and they were able to follow a structured home-based rehabilitation programme. This postoperative rehabilitation was self-directed and included passive ROM exercises and strengthening of the isometric quadriceps. Upon discharge, all patients were prescribed the same home medication regimen for 2 weeks, consisting of 90 mg of etoricoxib once daily, acetaminophen with codeine every 8 h, 75 mg of pregabalin at bedtime, 100 mg of aspirin once daily and 20 mg of omeprazole once daily. Follow-up visits were scheduled at 2 and 6 weeks and 3 and 6 months postoperatively. The length of inpatient stay was recorded for all patients and included as a secondary outcome measure.

### Data collection and outcome measurements

The primary outcome was peak isometric QS recovery, expressed as a percentage change from the preoperative value. The secondary outcomes were the Modified Timed Up and Go Test (MTUGT) score, serum inflammatory marker levels (CRP and erythrocyte sedimentation rate [ESR]), knee ROM, perioperative morphine consumption, peak postoperative blood glucose levels, number of insulin injections, pain scores using the Visual Analogue Scale (VAS) and functional outcomes assessed by the Western Ontario and McMaster Universities Osteoarthritis Index (WOMAC) and the Knee Society Score (KSS).

Baseline data included demographic characteristics, including age, sex, body mass index and comorbidities; preoperative laboratory values, including ESR and CRP; radiographic grading of osteoarthritis based on the Kellgren–Lawrence classification [12]; preoperative deformity; QS; MTUGT score; VAS score; ROM; and knee function scores, including WOMAC and KSS. Intraoperative and perioperative variables included quadriceps incision length, tourniquet time and estimated blood loss. Postoperative assessments were conducted on days 1 and 3 and at 2 weeks, 6 weeks, 3 months and 6 months. Isometric QS was measured using a digital hand-held dynamometer (MicroFET2^TM^, Hoggan Health Industries, Draper, UT, USA) following the standardised measurement protocol described by Wongsak et al.[13] MTUGT was defined as the time taken to rise from a chair and walk 3 m with or without gait assistance, as described in previous studies [13, 14].

Inflammatory markers (CRP and ESR) and ROM were evaluated at the same time points. Functional scores (WOMAC and KSS) were evaluated preoperatively and at 6 weeks, 3 months and 6 months postoperatively. Furthermore, IV or PA corticosteroid-related complications, such as wound or prosthetic joint infection, perioperative hyperglycaemia, prepatellar haematoma and steroid-associated complications, such as avascular necrosis and gastrointestinal bleeding, were recorded. All outcomes were assessed by the primary author, who was blinded to the group allocation and was not involved in the randomisation process. Adverse events and complications were systematically monitored by the research assistant and attending surgeon twice daily during hospitalization through structured checklists and spontaneous reporting until discharge and reassessed at each scheduled follow-up visit.

### Statistical analyses and sample size calculation

Data analyses were performed using STATA version 18 (StataCorp LLC, College Station, TX, USA). Continuous variables were presented as means ± standard deviations for normally distributed data and medians with interquartile ranges (IQR) for non-normally distributed data and compared using Student’s *t* test. Categorical variables were presented as proportions and compared using Fisher’s exact test or the chi-square test. An adjusted mixed-effects model was used to assess the statistical differences in postoperative outcomes, including QS recovery, VAS score, MTUGT score, number of morphine requests, ROM, WOMAC score and KSS. A modified intention-to-treat (mITT) analysis was performed, in which all randomized participants were included except for one patient who was excluded due to an unrelated traumatic event during follow-up. A two-sided *p* value < 0.05 indicated statistical significance.

The sample size was determined on the basis of data from a similar study. Assuming a type I error rate of 0.05, a dropout rate of 10%, a power of 80% and a standard deviation of 1.5, a sample size of 28 patients per group was required.

## Results

### General characteristics of the study population

**Fig. 1:**
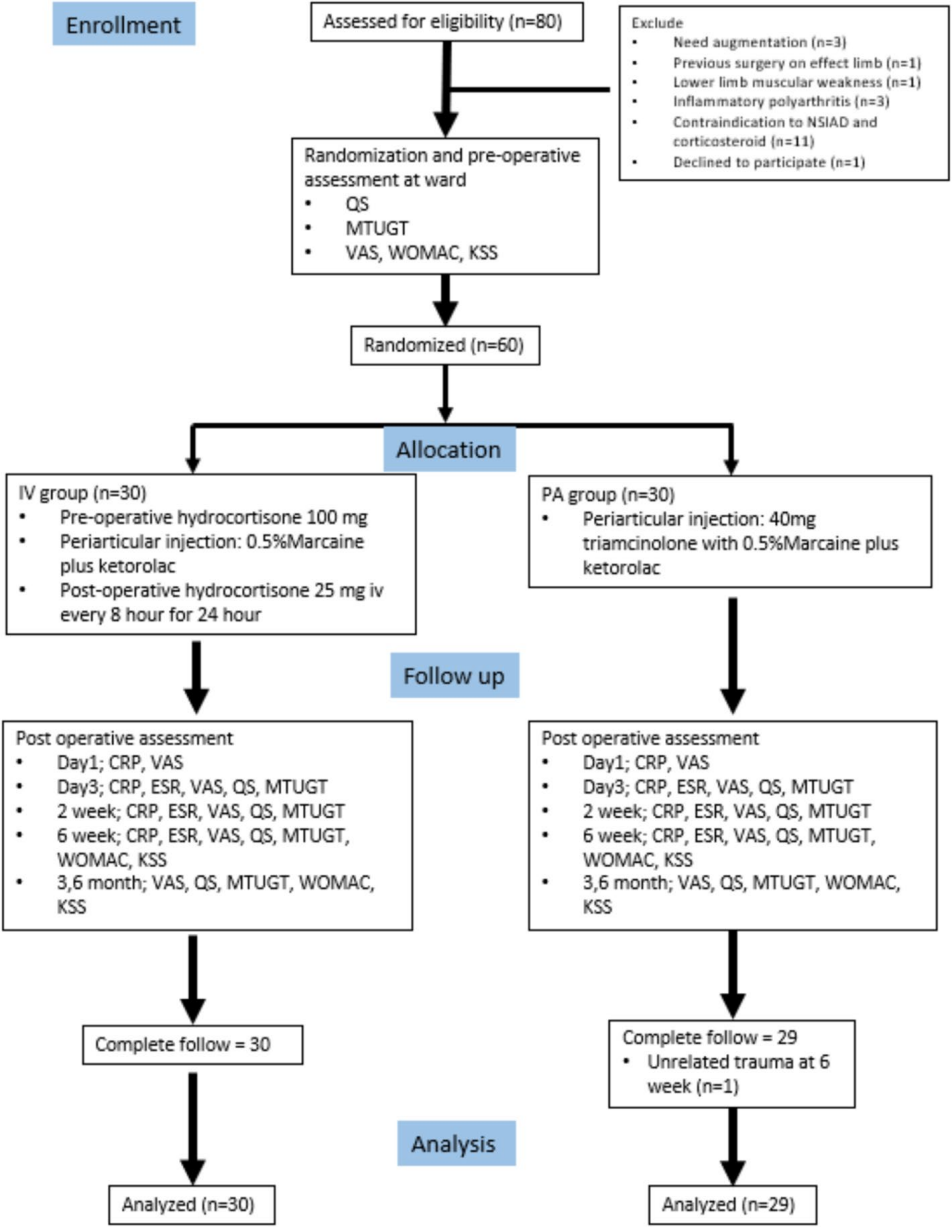
Randomization flow chart

A total of 80 patients were enrolled in this study (Fig. 1). Of the 80 patients, 20 were excluded due to the need for augmentation (*n* = 3), previous surgery on the affected limb (*n* = 1), lower limb muscular weakness (*n* = 1), inflammatory polyarthritis (*n* = 3), contraindications to nonsteroidal anti-inflammatory drugs or corticosteroids (*n* = 11) and refusal to participate (*n* = 1). The remaining 60 patients were included in this study and randomised into two equal groups: the IV group (*n* = 30) and the PA group (*n* = 30). All patients in the IV group completed the study and were included in the final analysis. One patient in the PA group was excluded owing to an unrelated traumatic event at the 6-week follow-up, resulting in 29 patients being included in the final analysis. Therefore, a modified intention-to-treat (mITT) analysis was performed.

The baseline demographic characteristics of the patients were comparable between the IV and PA groups, without significant differences in age, sex, body mass index, comorbidities or preoperative laboratory values (Table 1). However, a statistically significant difference in the side of surgery was observed between the two groups, with the IV group exhibiting a higher proportion of right-sided procedures than the PA group (80% vs 40%, *p* < 0.01). Additionally, no significant differences in intraoperative variables, including tourniquet duration, estimated intraoperative blood loss and length of quadriceps incision, were observed between the IV and PA groups.

**Table 1:** Demographic data

Variables	IV group (*n* = 30)	PA group (*n* = 29)	*p* value
Age^a^, year	70.73 ± 5.72	69.3 ± 7.36	0.40
Female gender (%)	26 (86.67)	28 (96.55)	0.35
Right side (%)	24 (80.0)	12 (40.0)	< 0.01*
BMI^a^	27.64 ± 3.47	26.94 ± 4.34	0.49
Comorbidities, number of patients (%)			
Diabetes	8 (26.67)	9 (30.00)	0.77
Hypertension	25 (83.33)	23 (76.67)	0.52
Cardiovascular	1 (3.33)	1 (3.33)	1.00
Preoperative serology			
Hematocrit^a^, %	40.23 ± 3.94	39.37 ± 3.59	0.380
Haemoglobin^a^, g/dL	13.05 ± 1.40	12.55 ± 1.22	0.15
Absolute neutrophil^b^, cells/µL	3536 (3014,4611)	3376 (3042,3967)	0.58
Absolute lymphocyte^a^, cells/µL	2161.5 ± 648.25	2202 ± 781.90	0.83
Platelet^b^, × 1000/mm^3^	259.5 (209,291)	262.5 (244,322)	0.17
Albumin^b^, g/dL	40 (37.9,41.9)	39.3 (38.3,40.9)	0.39
GFR^a^, ml/min	86.76 ± 11.71	85.62 ± 13.02	0.72
Glucose^b^, mg/dL	114.5 (107,133)	117.5 (107,123)	0.89
INR^a^	1.01 ± 0.05	1.01 ± 0.06	0.61
Varus OA knee (%)	29 (96.7)	26 (89.6)	0.35
OA grading by Kellgren–Lawrance, number of patients (%)			
3	3 (10)	1 (3.4)	1.00
4	27 (90)	28 (96.6)	
Intra-operative data			
Tourniquet time^a^, min	113.63 ± 14.06	107.07 ± 18.42	0.13
Estimate blood loss^b^, ml	30 (20,50)	30 (20,40)	0.82
Quadriceps cut^a^, cm	4.56 ± 0.38	4.46 ± 0.42	0.44

*IV* intravenous, *PA* periarticular, *BMI* body mass index, *GFR* glomerular infiltration rate, *INR* international normalized ratio, *OA* osteoarthritis, *yr* year, *g* gram, *dL* deciliter, *ml* microliter, *µL* microliter, *mm* millimeter, *mg* milligram, *min* minute, *cm* centimeter

^a^Value presented as mean (standard deviation) and calculated with unpaired *t* test

^b^Value presented as median (interquartile range) and calculated with Mann–Whitney *U* test

*Significant difference with *p* value < 0.05

### QS and MTUGT

On postoperative day 3, QS was significantly better preserved in the PA group than in the IV group (Table 2). The mean QS reduction on day 3 was −63.74% ± 17.67% in the IV group and −43.11% ± 23.36% in the PA group, with a statistically significant between-group difference of 20.63% (95% CI, 2.66–38.59; *p* = 0.02). Although the PA group consistently demonstrated a trend towards better QS recovery at all subsequent time points (from 2 weeks to 6 months), the differences were not statistically significant. At 6 months postoperatively, QS had increased in both groups, without a significant difference between the two groups (42.40% ± 46.37% in the IV group versus 50.22% ± 62.61% in the PA group; *p* = 0.39).

**Table 2:** VAS, QS, MTUGT, WOMAC and KSS scores

Perioperative outcomes	IV group	PA group	IV group	PA group	Mean different (95%CI)	*p* value
	mean ± SD	mean ± SD	Ls means (95%CI)	Ls means (95%CI)		
VAS						
(1) preop	6.10 ± 2.16	5.43 ± 1.55	6.10 (5.32,6.88)	5.43 (4.88,5.98)	−0.67 (−1.62,0.28)	0.17
(2) day 3	3.66 ± 1.91	3.53 ± 1.50	3.65 (2.96,4.34)	3.53 (3.00,4.07)	−0.12 (−0.99,0.75)	0.79
(3) 2 weeks	1.62 ± 1.80	1.14 ± 1.18	1.61 (0.96,2.26)	1.14 (0.71,1.57)	−0.47 (−1.25,0.31)	0.24
(4) 6 weeks	1.13 ± 1.28	1.17 ± 1.49	1.13 (0.68,1.59)	1.17 (0.63,1.71)	0.04 (−0.66,0.74)	0.91
(5) 3 months	0.33 ± 0.76	0.72 ± 0.92	0.33 (0.06,0.60)	0.72 (0.39,1.06)	0.39 (−0.04,0.82)	0.07
(6) 6 months	0.25 ± 0.59	0.36 ± 1.10	0.24 (0.02,0.45)	0.37 (−0.04,0.77)	0.13 (−0.33,0.58)	0.6
Change at day 3			−2.45 (−3.53,−1.37)	−1.9 (−2.61,−1.19)	0.55 (−0.73,1.83)	0.4
Change at 2 weeks			−4.49 (−5.27,−3.71)	−4.29 (−5.06,−3.52)	0.20 (−0.89,1.29)	0.72
Change at 6 weeks			−4.97 (−5.81,−4.13)	−4.26 (−4.99,−3.53)	0.71 (−0.39,1.81)	0.21
Change at 3 months			−5.77 (−6.52,−5.02)	−4.71 (−5.38,−4.04)	1.06 (0.06,2.06)	0.04*
Change at 6 months			−5.87 (−6.60,−5.14)	−5.07 (−5.75,−4.39)	0.80 (−0.19,1.79)	0.12
*p* value within period (1 versus 2), (1 versus 3), (1 versus 4), (1 versus 5), (1 versus 6)			< 0.01* all	< 0.01* all		
QS (present as percentage change from preoperative value)						
(1) Preop base line (newtons)	169.87 ± 52.01	148.80 ± 45.82	171.84 (150.33,193.35)	146.75 (127.04,166.45)	−25.09 (−54.12,3.94)	0.09
(2) day 3	−63.74 ± 17.67	−43.11 ± 23.36	−63.74 (−76.44,−51.04)	−43.11 (−55.82,−30.41)	20.63 (2.66,38.59)	0.02*
(3) 2 weeks	−46.29 ± 26.53	−30.90 ± 31.18	−46.29 (−58.99,−33.58)	−30.90 (−43.60,−18.20)	15.39 (−2.58,33.35)	0.09
(4) 6 weeks	−5.06 ± 27.98	5.26 ± 43.69	−5.06 (−17.77,7.64)	5.25 (−7.59,18.09)	10.32 (−7.74,28.38)	0.26
(5) 3 months	24.07 ± 38.70	28.85 ± 55.44	24.07 (11.37,36.77)	28.84 (16.01,41.69)	4.78 (−13.28,22.84)	0.60
(6) 6 months	42.40 ± 46.37	50.22 ± 62.61	42.40 (29.69,55.10)	50.21 (37.37,63.05)	7.82 (−10.25,25.88)	0.39
Change at day 3			−163.74 (−174.63,−152.86)	−143.11 (−157.56,−128.67)	20.63 (2.56,38.69)	0.02*
Change at 2 weeks			−146.29 (−157.17,−135.40)	−130.90 (−145.35,−116.45)	15.39 (−2.68,33.45)	0.09
Change at 6 weeks			−105.06 (−115.95,−94.18)	−94.75 (−109.35,−80.14)	10.32 (−7.84,28.48)	0.11
Change at 3 months			−75.93 (−86.82,−65.05)	−71.15 (−85.76,−56.55)	4.78 (−13.39,22.94)	0.25
Change at 6 months			−57.60 (−68.49,−46.72)	−49.79 (−64.39,−35.19)	7.82 (−10.35,25.98)	0.23
*p* value within period (1 versus 2), (1 versus 3), (1 versus 4), (1 versus 5), (1 versus 6)			< 0.01* all	< 0.01* all		
MTUGT (present as percentage change from preoperative value)						
(1) Preop baseline (sec)	11.71 ± 5.99	13.41 ± 6.80	8.63 (−2.51,19.77)	17.15 (6.15,28.16)	8.52 (−6.88,23.93)	0.28
(2) 3 days	301.33 ± 206.54	153.30 ± 100.24	301.33 (272.85,329.80)	153.30 (124.82,181.77)	−148.03 (−188.30,−107.76)	< 0.01*
(3) 2 weeks	75.02 ± 100.02	51.74 ± 100.61	75.02 (46.54,103.49)	51.74 (23.26,80.21)	−23.28 (−63.55,16.99)	0.26
(4) 6 weeks	−10.41 ± 42.92	−16.81 ± 31.95	−10.41 (−38.89,18.07)	−17.04 (−45.93,11.85)	−6.63 (−47.20,33.94)	0.75
(5) 3 months	−35.51 ± 25.77	−35.15 ± 28.63	−35.51 (−63.98,−7.03)	−35.39 (−64.28,−6.49)	0.12 (−40.45,40.69)	0.99
(6) 6 months	−46.57 ± 20.94	44.39 ± 22.43	−46.57 (−75.04,−18.09)	−44.62 (−73.51,−15.72)	1.95 (−38.62,42.52)	0.93
Change at 3 days			201.33 (159.11,243.54)	53.30 (29.27,77.32)	−148.03 (−196.72,−99.35)	< 0.01*
Change at 2 weeks			−24.98 (−67.20,17.23)	−48.26 (−72.28,−24.24)	−23.28 (−71.96,25.41)	0.35
Change at 6 weeks			−110.41 (−152.63,−68.19)	−117.10 (−141.37,−92.82)	−6.63 (−55.56,42.30)	0.79
Change at 3 months			−135.51 (−177.72,−93.29)	−135.44 (−159.71,−111.17)	0.12 (−48.81,49.05)	0.99
Change at 6 months			−146.57 (−188.79,−104.35)	−144.67 (−168.95,−120.40)	1.95 (−46.98,50.88)	0.94
*p* value within period (1 versus 2), (1 versus 3), (1 versus 4), (1 versus 5), (1 versus 6)			< 0.01*, 0.246, < 0.01*, < 0.01*, < 0.01*	< 0.01 all		
WOMAC						
(1) preop	80.63 ± 19.64	80.63 ± 19.03	80.62 ( 75.62,85.62)	80.62 (75.78,85.46)	1.42 × 10^−14^ (−6.96,6.96)	1.000
(2) 6 weeks	48.75 ± 12.32	49.93 ± 14.78	48.87 (44.21,53.52)	46.83 (40.97,52.69)	−2.03 (−9.51,5.45)	0.6
(3) 3 months	39.1 ± 10.02	39.75 ± 11.66	39.09 (35.10,43.08)	39.74 (35.46,44.03)	0.66 (−5.20,6.52)	0.83
(4) 6 months	32.32 ± 9.87	33.96 ± 12.99	32.39 (27.76,37.02)	33.93 (29.35,38.51)	1.54 (−4.96,8.05)	0.64
Change at 6 weeks			−31.87 (−39.63,−24.11)	−33.77 (−42.90,−24.64)	−2.03 (−13.93,9.87)	0.79
Change at 3 months			−41.53 (−49.29,−33.78)	−40.83 (−48.04,−33.62)	0.66 (−9.87,11.19)	0.9
Change at 6 months			−48.30 (−57.01,−39.59)	−46.46 (−53.56,−39.36)	1.54 (−9.71,12.80)	0.79
*p* value within period (1 versus 2), (1 versus 3), (1 versus 4)			< 0.01* all	< 0.01* all		
KSS						
(1) preop	105.3 ± 23.49	99.86 ± 26.02	104.57 (99.65,109.49)	101.35 (95.71,106.99)	−3.22 (−10.71,4.27)	0.4
(2) 6 weeks	130.83 ± 19.16	136.69 ± 25.05	129.80(122.66,136.94)	137.56 (129.08,146.05)	7.77 (−3.27,18.80)	0.17
(3) 3 months	159.13 ± 14.98	153.67 ± 21.05	158.40(152.09,164.72)	155.76 (148.03,163.49)	−2.64 (−12.60,7.32)	0.6
(4) 6 months	168.61 ± 10.38	165.30 ± 18.29	166.70(161.94,171.47)	165.61 (159.06,172.16)	−1.09 (−9.09,6.90)	0.79
Change at 6 weeks			25.32 (15.46,35.18)	36.60 (26.23,46.98)	10.99(−3.30,25.28)	0.13
Change at 3 months			53.83 (43.89,63.77)	54.18 (43.65,64.70)	0.58(−13.88,15.05)	0.94
Change at 6 months			62.98 (54.15,71.82)	64.07 (54.57,73.57)	2.13(−10.52,14.78)	0.74
*p* value within period (1 versus 2), (1 versus 3), (1 versus 4)			< 0.01* all	< 0.01* all		

Comparison of VAS, QS, MTUGT, WOMAC and KSS scores between the IV-group and PA-group

*IV* intravenous, *PA* periarticular, *SD* standard deviation, *Ls means* least square means, *CI* confident interval, *VAS* visual analog pain scale, *QS* quadriceps strength, *MTUGT* modified time up and go test, *WOMAC* Western Ontario and Mcmaster Universities Osteoarthritis Index, *KSS* Knee Society Score

**Fig. 2:**
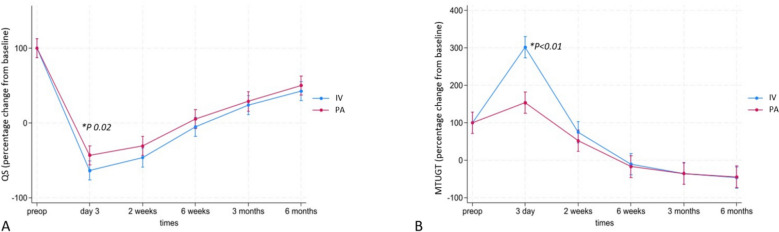
QS and MTUGT. Line graphs showing the percentage change from the preoperative baseline in mean quadriceps strength (**A**) and mean Modified Timed Up and Go Test (MTUGT) performance (**B**). The PA group exhibited significantly less reduction in quadriceps strength and faster MTUGT times on postoperative day 3 compared with the IV group

On postoperative day 3, the IV group demonstrated a greater increase in MTUGT scores from baseline (301.3%, 95% CI 272.9–329.8) than the PA group (153.3%, 95% CI 124.8–181.8), with a significant mean difference of −148.0% (95% CI −188.3 to −107.8; *p* < 0.01) (Fig. 2). No statistically significant differences were observed between the groups at 2 and 6 weeks and 3 and 6 months postoperatively. At 6 months postoperatively, both groups showed improvements from baseline (IV: −46.6%, 95% CI −75.0 to −18.1; PA: −44.6%, 95% CI −73.5 to −15.7), without a significant difference between the two groups (mean difference: 2.0%, 95% CI −47.0 to 50.9; *p* = 0.93).

### Postoperative pain and inflammatory markers (VAS, morphine, ESR and CRP)

**Fig. 3:**
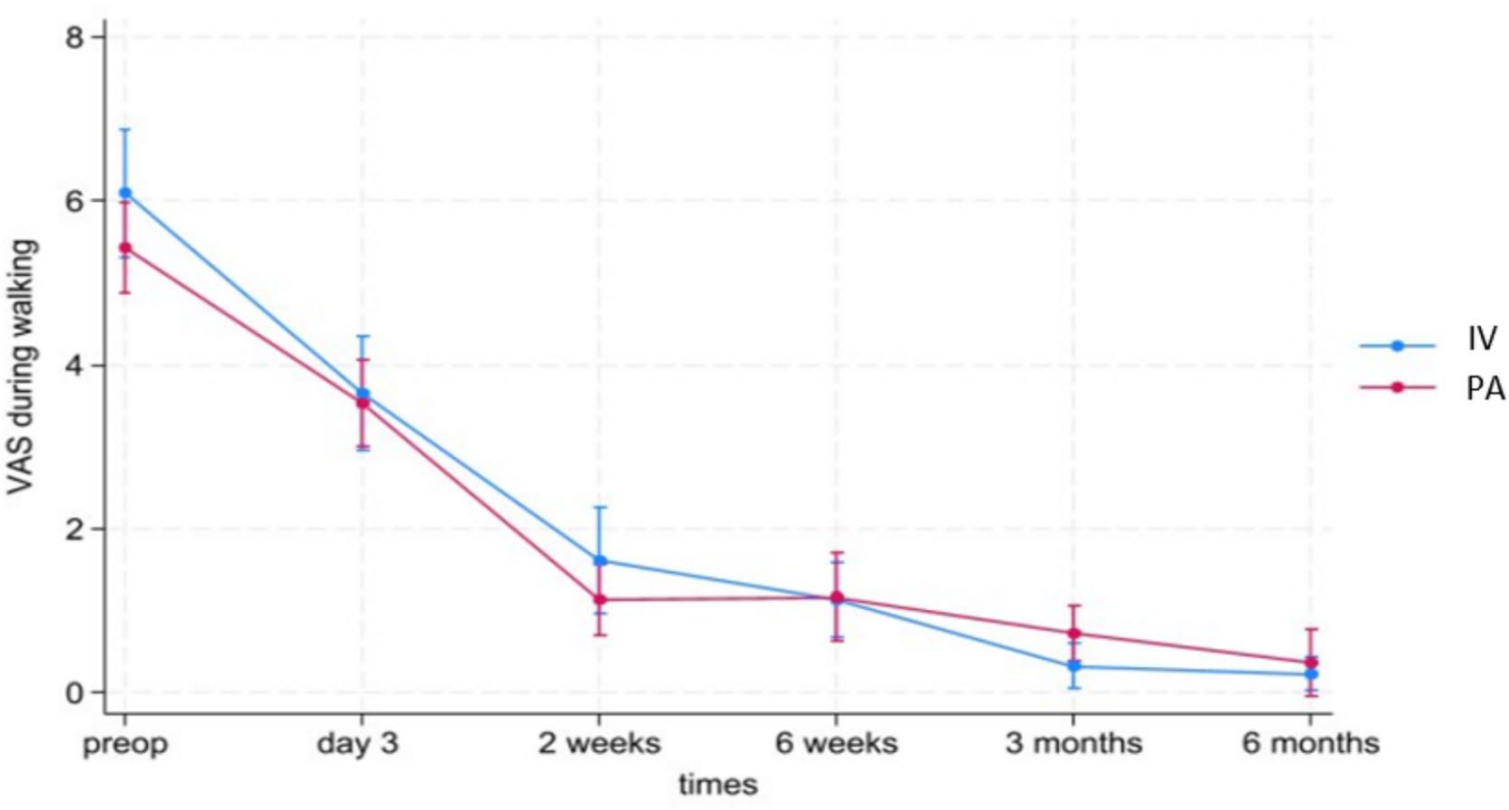
VAS scores. Line graph showing mean Visual Analog Scale (VAS) pain scores from the perioperative period through 6 months of follow-up

The baseline VAS scores were slightly higher in the IV group than in the PA group. However, the difference was not significant (Table 2). Both groups showed significant pain reduction at all-time points compared with the preoperative score, without a significant difference between the two groups. However, the IV group showed a significantly greater improvement in VAS scores than the PA group at 3 months postoperatively (mean difference from baseline: 1.06; 95% CI 0.06–2.06, *p* = 0.04) (Fig. 3).

The postoperative morphine use was comparable between the two groups at all-time points (Table 3). On day 0, 83.3% and 80.0% of patients in the IV and PA groups, respectively, did not require morphine (*p* = 1.00). Similar patterns were observed on day 1 (76.7% versus 73.3%; *p* = 0.22), day 2 (80.0% versus 93.3%; *p* = 0.30) and day 3 (93.3% in both groups; *p* = 1.00). Similarly, the use of ondansetron was comparable between the two groups (Table 3), with 6 of 29 patients (20.7%) in the PA group and 5 of 30 patients (16.7%) in the IV group requiring antiemetic treatment (*p* = 0.75; risk ratio 1.24, 95% CI 0.43–3.62), indicating no significant difference in postoperative nausea management between groups.

**Table 3:** Morphine consumption and ondansetron use

Total morphine injection	Times	IV group (%, *n* = 30)	PA group (%, *n* = 29)	*p* value
Day 0	0	25 (83.3)	24 (80)	1.00
	1	5 (16.7)	5 (16.7)	
	2	0 (0)	1 (3.3)	
Day 1	0	23 (76.7)	22 (73.3)	0.22
	1	4 (13.3)	8(26.7)	
	2	2 (6.7)	0(0)	
	4	1 (3.3)	0(0)	
Day 2	0	24 (80)	28 (93.3)	0.30
	1	4 (13.3)	2 (6.7)	
	2	2 (6.7)	0 (0)	
Day 3	0	28 (93.3)	28 (93.3)	1.00
	1	2 (6.7)	2 (6.7)	
Ondansetron use		5 (16.7)	6 (20.7)	0.75

*IV* intravenous, *PA* periarticular, *n* number

Comparison of postoperative morphine consumption and ondansetron use between the IV and PA groups

Regarding postoperative inflammatory markers, CRP levels were significantly lower in the PA group than in the IV group during the early postoperative period (day 1: *p* = 0.01; day 3: *p* < 0.01; 2 weeks: *p* = 0.01) (Table 4). However, no significant differences in ESR were observed between the two groups at any time point, despite a trend towards higher values in the PA group at later follow-up (6 weeks: *p* = 0.09).

**Table 4:** Glucose ESR CRP ROM

Perioperative outcomes	IV group (*n* = 30)	PA group (*n* = 29)	*p* value
Hematocrit^a^ (%)			
Preop	40.23 ± 3.94	39.37 ± 3.59	0.38
Day 3	35.79 ± 4.65	33.93 ± 4.28	0.11
2 week	37.15 ± 3.06	36.74 ± 2.92	0.61
Glucose^b^ (mg/dL)			
Preop	114.5 (107,133)	117.5 (109,123)	0.93
Day 1	156.5 (134,191)	161 (139,177)	0.76
Day 3	106 (93,122)	115 (106,127)	0.02*
2 weeks	112 (99.5,117.5)	102 (95,113)	0.28
Insulin^b^ (number of injections)			
Day 1	0 (0,4)	0 (0,0)	0.48
Day 2	0 (0,0)	0 (0,0)	0.15
Day 3	0 (0,0)	0 (0,0)	1.00
ESR (mm/hr.)			
Preop^b^	20 (15,33)	27.5 (12,36)	0.73
Day 3^a^	40.03 ± 15.37	54.1 ± 22.20	0.31
2 weeks^b^	42 (31,61)	36 (30,53)	0.38
6 weeks^a^	31.86 ± 15.63	39.89 ± 19.20	0.09
CRP^b^ (mg/dL)			
Preop	2.25 (1.3,3.3)	1.3 (1,4.2)	0.25
Day 1	15.45 (9.4,28.6)	10.4 (5.3,17.2)	0.01*
Day 3	119.05 (69.6,146.1)	65.45 (38.3,96)	< 0.01*
2 weeks	8.9 (5.6,14.2)	3.9 (1.65,9.6)	0.01*
6 weeks	4.6 (3.1,6.1)	3.25 (1.4,6.6)	0.44
Maximum knee flexion^a^ (degrees)			
Preop	115.07 ± 17.61	119.23 ± 13.56	0.31
Day 3	82.59 ± 10.94	91.0 ± 11.03	< 0.01*
2 weeks	97.41 ± 8.68	103.22 ± 12.16	0.04*
6 weeks	112.16 ± 10.69	116.72 ± 9.40	0.09
3 months	119.0 ± 14.30	121.38 ± 8.52	0.44
6 months	119.39 ± 10.29	122.79 ± 8.19	0.18

*IV* intravenous, *PA* periarticular, *INR* international normalized ratio, *hr.* hour, *dL* deciliter, *mm* millimeter, *mg* milligram

^a^Value presented as mean (standard deviation) and calculated with unpaired *t* test

^b^Value presented as median (interquartile range) and calculated with Mann–Whitney *U* test

*Significant different with *p* = value < 0.05

Comparison of glucose, ESR, CRP and ROM between the IV-group and PA-group

### Functional knee score and knee ROM

**Fig. 4:**
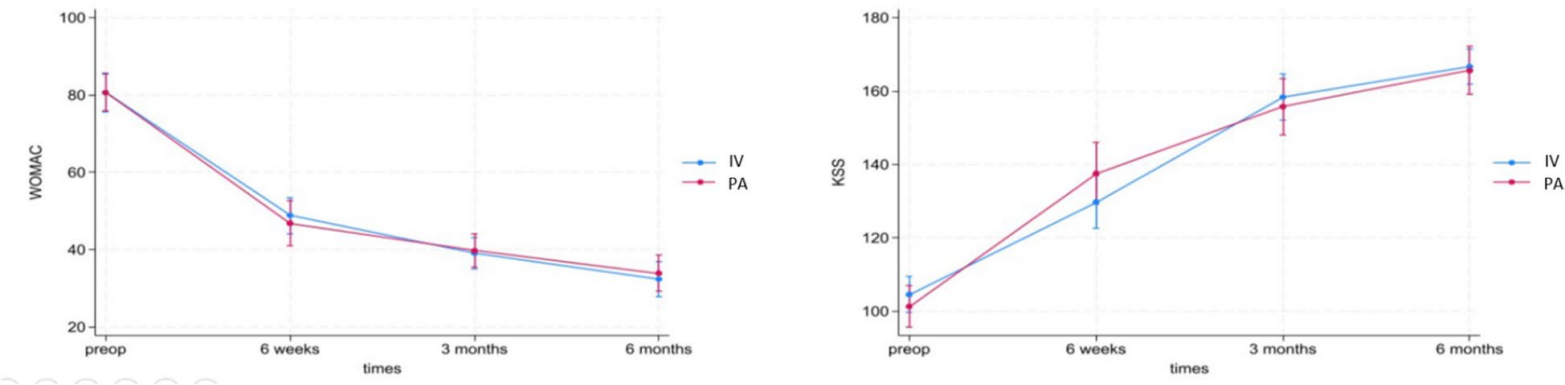
WOMAC and KSS scores. Line graphs showing changes in knee function scores: WOMAC knee score (**A**) and Knee Society Score (**B**). Both groups demonstrated significant improvement in functional scores from the preoperative period to 6-month follow-up; however, no significant differences were observed between the groups

Both groups demonstrated significant improvements in WOMAC and KSS scores over time (*p* < 0.01), without statistically significant differences between the two groups at any follow-up interval (Fig. 4). The PA group demonstrated a trend towards greater improvement in KSS at 6 weeks. However, it did not reach statistical significance.

Preoperative maximum knee flexion was similar between the IV and PA groups (115.1° ± 17.6° versus 119.2° ± 13.6°, *p* = 0.31) (Table 4). The PA group demonstrated superior early recovery compared with the IV group, with significantly greater flexion on day 3 (91° ± 11° versus 82.6° ± 10.9°, *p* < 0.01) and at week 2 (103.2° ± 12.2° versus 97.4° ± 8.7°, *p* = 0.04). However, the differences became nonsignificant by 6 weeks (PA: 116.7° versus IV: 112.2°, *p* = 0.09) and remained comparable at 3 and 6 months (all *p* > 0.18).

### Hyperglycaemia and complications

Preoperative glucose levels were comparable between the IV (114.5 mg/dL, IQR: 107–133) and PA (117.5 mg/dL, IQR: 109–123) groups (*p* = 0.93) (Table 4). Both groups exhibited a transient postoperative rise in glucose levels, peaking on day 1 (IV: 156.5 mg/dL, IQR: 134–191 versus PA: 161 mg/dL, IQR: 139–177, *p* = 0.76), without significant differences in insulin requirements (*p* > 0.15). On postoperative day 3, the PA group demonstrated higher glucose levels (115 mg/dL, IQR: 106–127 versus IV: 106 mg/dL, IQR: 93–122, *p* = 0.02). However, this did not increase the insulin requirement (*p* = 1.00). No significant differences were observed between the two groups beyond day 3 (all *p* > 0.28). No postoperative wound or periprosthetic joint infections were observed in either group.

## Discussion

Corticosteroid administration during the perioperative period of TKA has been widely recognised for its anti-inflammatory and analgesic benefits. It significantly reduces postoperative pain, mitigates nausea and vomiting and facilitates early mobilisation and rehabilitation [3, 4]. IV and PA injection are two commonly used primary administration routes, each with distinct pharmacokinetics and tissue distribution [7, 15]. Both approaches aim to mitigate the inflammatory response and enhance functional recovery. However, the comparative effectiveness of these two administration routes has rarely been investigated.

In this study, hydrocortisone was selected as the corticosteroid agent owing to its broader glucocorticoid and mineralocorticoid activity profile compared with dexamethasone, which has a longer half-life but limited mineralocorticoid effects [16, 17]. Unlike dexamethasone, hydrocortisone has the same structure as natural cortisol and more closely mimics the endogenous stress response, thereby reducing the risk of hypothalamic–pituitary–adrenal axis suppression with repeated dosing [17, 18]. Previous research has demonstrated that the use of hydrocortisone during major orthopaedic procedures can effectively control inflammation while minimising prolonged immunosuppression. These pharmacologic properties support the use of hydrocortisone as a viable agent for perioperative modulation of inflammation in TKA [5, 19]. Furthermore, its relatively short half-life allows for rapid systemic clearance, thereby limiting prolonged corticosteroid exposure. These characteristics may reduce the risk of corticosteroid-related adverse effects, such as sustained hyperglycaemia, impaired wound healing and adrenal suppression [17]. Importantly, this trial compared both the route and type of corticosteroid administered. Hydrocortisone and triamcinolone acetonide differ in glucocorticoid potency, half-life and tissue distribution, which may partly explain the observed differences in early quadriceps strength recovery. Triamcinolone provides sustained local anti-inflammatory activity, whereas hydrocortisone has a shorter duration and broader mineralocorticoid profile. Therefore, part of the effect may reflect pharmacologic differences between agents as well as the route of administration.

The total hydrocortisone dosing regimen used in this study was 175 mg per day, aligning with current recommendations for perioperative stress-dose corticosteroid coverage in noncomplex surgical procedures. Based on endocrine and perioperative management guidelines, hydrocortisone doses of 50–150 mg per day are considered adequate for moderate- to high-stress procedures, including TKA, in patients without chronic steroid use or adrenal insufficiency [16, 17]. Therefore, the selected dosing regimen is considered appropriate for perioperative use in patients with noncomplex primary TKA. The PA group received a single dose of 40 mg of triamcinolone acetonide, which has a higher glucocorticoid potency than hydrocortisone, as 1 mg of triamcinolone is approximately equivalent to 5 mg of hydrocortisone [20]. Thus, 40 mg of triamcinolone corresponds to approximately 200 mg of hydrocortisone, making it comparable to or slightly higher than the total hydrocortisone dose used in the IV group.

In this study, both IV and PA corticosteroid injection resulted in substantial postoperative pain relief, as evidenced by significant improvements in VAS scores across all time points compared with baseline. Additionally, no statistically significant differences in VAS scores or total morphine requirements were observed between the two groups at any time point. These findings are consistent with those of previous studies that reported that corticosteroids effectively reduce pain and opioid use in the early postoperative period [7, 21–23]. The comparable analgesic outcomes indicate that both PA and IV corticosteroid administration are beneficial for perioperative pain management in patients undergoing TKA, offering clinicians flexible options based on patient-specific considerations.

The recovery of QS and functional mobility, measured by the MTUGT, showed early advantages in the PA group. On postoperative day 3, the PA group demonstrated significantly better QS preservation and shorter MTUGT times than the IV group. A between-group difference of approximately 20% in early postoperative QS may be clinically meaningful, given the established relationship between quadriceps power, knee stability and gait performance in the early rehabilitation phase of TKA. A trend towards improved QS in the PA group persisted at 2 weeks postoperatively. These functional improvements may be partly due to reduced local inflammation, as indicated by significantly lower serum CRP levels in the PA group from day 1 to week 2. The reduced inflammation might have resulted in less knee swelling and pain, facilitating earlier ambulation and promoting muscle recovery. In addition, decreased periarticular swelling and joint effusion may further enhance quadriceps function by reducing arthrogenic muscle inhibition (AMI), a reflex mechanism that limits voluntary muscle activation[24–26]. Even small amounts of intra-articular fluid have been shown to impair neuromuscular activation and reduce quadriceps strength. Therefore, the reduction of joint effusion may improve motor unit recruitment and the mechanical efficiency of muscle contraction, contributing to improved early functional. These findings are consistent with those of previous studies, which showed that PA corticosteroid administration yielded superior perioperative pain control compared with IV corticosteroid administration, as evidenced by lower VAS scores.[22, 23, 27] To the best of our knowledge, this is the first study to demonstrate objective-measurable evidence that PA corticosteroid administration has a superior effect of enhancing early postoperative QS recovery and accelerating postoperative walking speed.

In this study, the PA group exhibited greater maximum knee flexion on postoperative days 3 and 14. This finding differs from that of Qingtian et al., who reported no significant difference in ROM between PA and IV administration [23]. We hypothesise that the improved early knee flexion observed in our study may be due to better early pain control, reduced PA oedema and enhanced joint mobility. Previous studies have shown that targeted PA corticosteroid injections can yield superior functional outcomes when delivered directly to inflamed tissues [22, 28]. Despite these early functional improvements, no significant differences were observed in outcome measures beyond 6 weeks. Particularly, the WOMAC and KSS scores at 3 and 6 months were comparable between the two groups, indicating that the benefits of PA corticosteroid administration may be limited to the early postoperative period. These findings are consistent with those of most previous studies [7, 28].

The absence of a sustained benefit beyond the immediate postoperative period in the IV group may be attributed to the rapid systemic clearance of hydrocortisone, resulting in limited tissue-level persistence after administration. Conversely, the PA triamcinolone injection delivers a depot formulation directly to the surgical site, thereby allowing for prolonged local anti-inflammatory activity [29, 30]. This presence of localised and sustained drug may account for the continued functional advantages observed in the PA group up to 2 weeks postoperatively.

Regarding safety, both delivery methods were associated with transient perioperative hyperglycaemia, without significant differences in insulin requirements or the incidence of sustained hyperglycaemia. Notably, the PA group had a higher glucose level on postoperative day 3, highlighting the importance of routine blood glucose monitoring regardless of the corticosteroid delivery method. However, the absolute glucose levels remained within a near-normal clinical range and are unlikely to have a clinically meaningful impact on patient outcomes, including infection risk or wound healing. In this study, no wound complications, surgical site infections or periprosthetic joint infections were observed in either group, supporting previous evidence that perioperative corticosteroid use in TKA does not increase the risk of postoperative infections [31–33].

This study has several limitations. First, the sample size was relatively small. However, it was adequate to detect statistically significant differences in primary outcomes, such as QS and early functional recovery. Second, patient blinding was not possible due to the nature of the interventions. However, most outcomes, such as dynamometric strength testing, inflammatory markers and ROM, were objective and less susceptible to bias. Third, this study was conducted at a single academic centre with a standardized protocol and a single surgeon, which may limit the generalizability of the results to other surgical settings. In addition, the analysis was conducted using a modified intention-to-treat approach due to the exclusion of one patient with an unrelated traumatic event, which may introduce a minimal risk of attrition bias. Additionally, a statistically significant imbalance in the side of surgery between groups was observed. Although quadriceps strength recovery was expressed as a percentage change from each patient’s preoperative baseline on the same limb, which may partially mitigate the influence of limb dominance, residual confounding related to the dominant versus non-dominant limb cannot be entirely excluded. Furthermore, this study simultaneously compared both the route of administration and the corticosteroid agent. Therefore, it is not possible to definitively determine whether the observed superiority of the PA group was attributable to the local injection route or to the pharmacological properties of triamcinolone acetonide, such as its greater potency and longer half-life. Finally, the long-term effects of corticosteroid administration beyond 6 months were not evaluated and warrant further investigation.

## Conclusions

A single PA injection of 40 mg triamcinolone acetonide in patients undergoing primary TKA can provide superior early postoperative outcomes compared with an IV hydrocortisone regimen. The PA group showed significantly better QS recovery, improved functional mobility and greater knee flexion in the early postoperative period until 2 weeks postoperatively. These benefits may be related to enhanced local anti-inflammatory effects, resulting in reduced pain and swelling and facilitating early rehabilitation. Importantly, both corticosteroid regimens were well-tolerated, without increasing perioperative complications, such as transient hyperglycaemia, wound infection and prosthetic joint infection.

## Data Availability

De-identified individual participant data (IPD), data dictionary and statistical analysis code will be available from the corresponding author upon reasonable request, following approval by the institutional ethics committee and completion of a data-sharing agreement.

## Declarations

### Thai clinical trials registration number

TCTR20240419006 at thaiclinicaltrials.org.
